# Association between changes in adherence to the 24-hour movement guidelines with depression and anxiety symptoms among Chinese adolescents: a prospective population-based study

**DOI:** 10.1186/s13034-024-00836-7

**Published:** 2024-11-10

**Authors:** Herui Wu, Yi-fan Lin, Liwen Yang, Wenjian Lai, Yanzhi Li, Ye Xu, Wanxin Wang, Lei Yang, Ciyong Lu, Bin Yan

**Affiliations:** 1 Department of Medical Statistics and Epidemiology, School of Public Health, Sun Yat-Sen University, 74 Zhongshan Rd 2, Guangzhou, 510080 Guangdong China; 2 Department of Spine Surgery, the First Affiliated Hospital, Shenzhen University, Shenzhen, Guangdong China; 3 Department of Spine Surgery, Shenzhen Second People’s Hospital, Shenzhen, Guangdong China

**Keywords:** Physical activity, Screen time, Sleep duration, Depressive symptoms, Anxiety symptoms

## Abstract

**Background:**

The 24-hour movement guidelines (24-HMG) include screen time (ST), sleep duration, and physical activity. Previous studies have explored the associations of adherence to the 24-HMG with depression and anxiety symptoms among adolescents, ignoring changes in behaviors. This study aimed to examine whether changes in adherence to the 24-HMG were associated with depression and anxiety symptoms among adolescents.

**Methods:**

We recruited adolescents from Shenzhen, China in 2021 and followed them up 1 year later. Changes in adherence to the individual 24-HMG were categorized into four groups: persistent non-adherence, adherence to non-adherence, non-adherence to adherence, and persistent adherence. Changes in the numbers of adherence to the overall 24-HMG were obtained by subtracting the number of guidelines adhered to in wave 2 from that in wave 1.

**Results:**

We included 12,570 participants aged 9–18 years with 52.2% boys. Compared with persistent non-adherence for the ST/sleep duration guideline, non-adherence to adherence and persistent adherence were associated with lower depression and anxiety symptoms, but adherence to non-adherence was not. Changes in adherence to the physical activity guideline were not related to outcomes. The *β* coefficients (95% CIs) for each point increase in changes in the numbers of adherence to guidelines were − 0.58 (− 0.69, − 0.47) and − 0.43 (− 0.53, − 0.33) for depression and anxiety symptoms, respectively. The association of persistently adhering to sleep guideline with anxiety symptoms and the associations of changes in the numbers of adherence to the 24-HMG had sex differences.

**Conclusions:**

Maintaining and strengthening healthy movement behaviors to meet more guidelines of the 24-HMG, especially sleep and ST, may be beneficial for preventing depression and anxiety symptoms in adolescents.

**Supplementary Information:**

The online version contains supplementary material available at 10.1186/s13034-024-00836-7.

## Introduction

Depression and anxiety symptoms are common mental health problems among adolescents [1], with prevalence of 25.2% and 20.5%, respectively [2]. Numerous previous studies have found that insufficient movement behaviors (e.g., physical inactivity, excessive screen time (ST), and inadequate sleep duration) are potential risk factors for depression and anxiety symptoms [3]. The movement behaviors are interrelated components of the 24-h continuum and should be considered simultaneously when exploring their association with events [4]. The Canadian 24-Hour Movement Guidelines (24-HMG) recommend adolescents need at least 60 min of moderate-to-vigorous physical activity (MVPA) per day, no more than 2 h of ST per day, and 9–11 h of sleep per night (aged 5–13 years) or 8–10 h of sleep per night (aged 14–17 years) [5]. Previous studies have found that adherence to the 24-HMG is beneficial for preventing depression and anxiety symptoms among adolescents [3, 6, 7].

With increasing age, adolescents tend to exhibit a decline in physical activity levels, an increase in ST, and a reduction in sleep duration, and these trends may have a significant impact on their mental health [8, 9]. Capturing changes in adherence to the 24-HMG, rather than solely assessing them at baseline, holds significance as it reflects the associated risks adolescents face when their movement behaviors change in the real world. However, most previous studies relied on a singular measurement of the 24-HMG, ignoring dynamic changes in adherence to the 24-HMG [6, 7, 10]. To date, one longitudinal study from Canada has explored the associations of changes in adherence to the 24-HMG with depression symptoms in adolescents [11]. Another longitudinal study from the United States has assessed the correlation between changes in adherence to the overall 24-HMG from adolescence to adulthood and depression symptoms in adulthood [12]. In contrast, evidence remains lacking regarding the association of changes in adherence to the 24-HMG with anxiety symptoms in adolescents. Additionally, there are differences in movement behaviors between Western and Eastern adolescents. Specifically, the proportion of meeting the ST guideline exceeded most Western countries in China while the proportions of meeting physical activity and sleep guidelines were lower [6, 13]. These discrepancies may be attributed to the heavy emphasis on academic achievement in Chinese society [6]. Thus, the results for Western adolescents may not be generalizable to Eastern adolescents. It is necessary to explore the associations of changes in adherence to the 24-HMG with depression and anxiety symptoms among Eastern adolescents.

In addition, some previous studies have shown that the association between adherence to the 24-HMG and mental health among adolescents may differ according to sex [14, 15]. This is further supported by the fact that there were significant differences in the prevalence of depression and anxiety symptoms between boys and girls [16, 17]. Moreover, the proportion of boys who meet all 3 components of the 24-HMG is significantly higher than that of girls [15], and changes in adherence to the 24-HMG also show sex differences [18]. It suggests that different intervention options may be needed for adolescents of different sexes when preventing depression and anxiety symptoms. However, to our knowledge, no previous study has analyzed sex differences in changes in adherence to the 24-HMG in depression and anxiety symptoms.

Therefore, this study aimed to explore the associations of changes in adherence to the overall and individual 24-HMG with depression and anxiety symptoms among adolescents, as well as sex differences in the above associations.

## Methods

### Design and participants

This longitudinal study used data from a study on the construction of an adolescent scoliosis risk prediction model based on the screening cohort of primary and secondary school students in Shenzhen (ChiCTR2400081430), which annually collects scoliosis-related data since 2013 launched in Shenzhen, China. Further details about the study design have been published [19]. At baseline, we randomly selected 5 districts of Shenzhen (Bao’an, Luohu, Longgang, Longhua, and Futian District) and 6 primary schools, 10 junior high schools, and 4 senior high schools were randomly selected from each selected district. To reduce the rate of loss to follow-up in the subsequent year, students in primary schools (i.e., grades 4–5), junior high schools (i.e., grades 7–8), and senior high schools (i.e., grades 10–11) were invited to participate in the screening program voluntarily in 2021 (Wave 1). The follow-up survey was distributed approximately 12 months after baseline in 2022 (Wave 2). Participants completed a questionnaire so that information could be collected on a variety of demographics, physical activity, ST, sleep duration, depression, and anxiety symptoms in Wave 1 and Wave 2. This study was approved by the Shenzhen Second People’s Hospital Institutional Review Board (20211013002-fs01). Written informed consents were obtained from each participant who was at least 18 years old or from one of the parents of each participant who was under 18 years old.

**Fig. 1:**
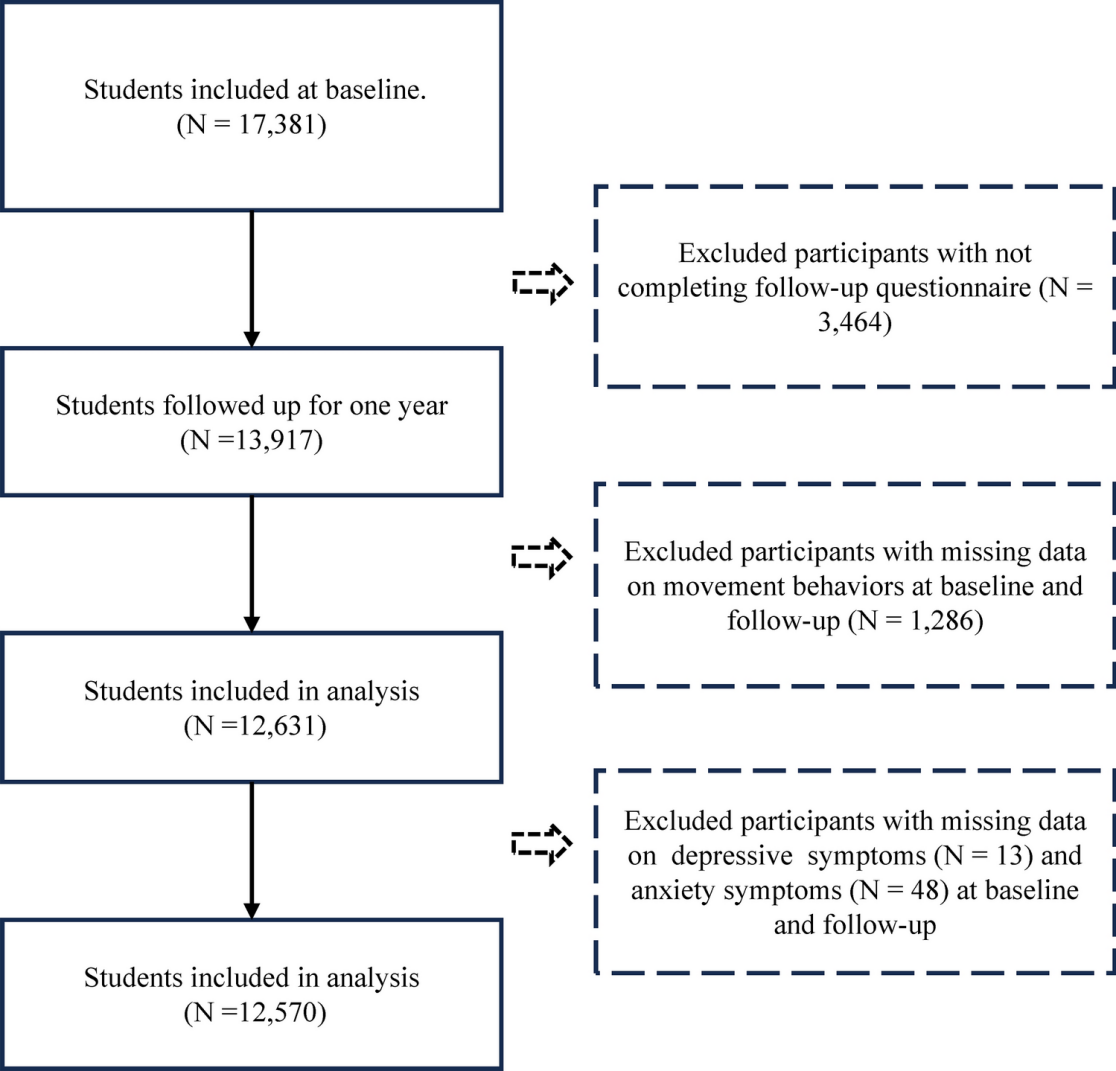
Flow of study participants

To explore the associations of changes in adherence to the 24-HMG with depression and anxiety symptoms, wave 1 was considered as the baseline, and data from wave 2 was used to evaluate depression and anxiety symptoms. Our final analysis included 13,917 participants in both wave 1 and wave 2. Of these participants, 3464 did not complete the follow-up survey, 1286 were missing data on movement behaviors, and 61 were missing data on depression and anxiety symptoms. After these exclusions, 12,570 participants were included in the statistical analysis (Fig. 1).

### Measures

#### Physical activity

In wave 1 and wave 2, physical activity was assessed using the International Physical Activity Questionnaire-Short Form (IPAQ-SF) [20]. Measurements of MVPA used four items from the IPAQ-SF that recorded the frequency (days) and duration (in minutes) of performing moderate (e.g., light-lifting, biking, double tennis) and vigorous (e.g., weight-lifting, aerobics) intensity activities [21]. Multiplication of frequency and duration scores resulted in an estimate of weekly hours invested in vigorous PA and moderate PA. According to the 24-HMG, meeting the physical activity recommendation requires that participants report 7 days with a minimum of 60 min of physical activity daily [5].

#### Screen time

In wave 1 and wave 2, ST was assessed using a question that asked students to report the average number of hours per day that they spent using computers, using smartphones (or tablets), watching television, and electronic goods (e.g., handheld game consoles, console games, etc.) on the weekday and weekend. Participants responded to each question using a 4-point scale: < 30 min, 30–60 min, 60–90 min, or > 90 min. ST on weekdays and weekends was weighted at a ratio of 5:2 to generate an average ST per day [6]. According to the 24-HMG, meeting the ST recommendation requires daily ST ≤ 2 h per day [5].

#### Sleep duration

In wave 1 and wave 2, sleep duration was measured using two items that asked students to self-report during the last week how many hours of sleep on weekdays and weekend days they usually get. Sleep duration on weekdays and weekend days was weighted at a ratio of 5:2 to generate an average sleep duration per day. According to the 24-HMG, meeting sleep recommendations requires 9–11 h of sleep for students aged 5–13 and 8–10 h of sleep for students aged 14–17 [5].

#### Changes in adherence to all components in the 24-hour movement guidelines

According to the physical activity status of adolescents in wave 1 and wave 2, changes in adherence to the physical activity guideline were divided into four categories: persistent non-adherence, adherence to non-adherence, non-adherence to adherence, and persistent adherence. Changes in adherence to ST and sleep guidelines were also grouped in the same way.

Changes in the numbers of adherence to the 24-HMG were obtained by subtracting the number of guidelines adhered to in wave 2 from that in wave 1 [22]. The possible total scores range from − 3 to 3, with a higher score indicating that adolescents met more guidelines in wave 2 compared to wave 1. The changes were divided into three categories: unchanged (scores = 0), increasing (scores > 0), and decreasing (scores < 0).

#### Depression symptoms

In wave 1 and wave 2, depression symptoms were measured via the Chinese version of the nine-item Patient Health Questionnaire (PHQ-9). The PHQ-9 instrument consists of 9 questions on depression symptoms over the last 14 days and is widely used in Chinese adolescents with good reliability and validity [23]. Each item of PHQ-9 ranges from 0 (not at all) to 3 (almost every day). The total score in wave 2 ranges from 0 to 27 with a higher score indicating more severe depression symptoms. Additionally, depression symptoms were defined as PHQ-9 scores ≥ 5 [24], analyzed as a dichotomous variable (yes or no), with Cronbach’s alpha coefficients of 0.88 and 0.91 in waves 1 and 2, respectively.

#### Anxiety symptoms

In wave 1 and wave 2, anxiety symptoms were measured using the Chinese version of the seven-item Generalized Anxiety Disorder Scale. The GAD-7 instrument consists of 7 questions on anxiety symptoms over the last 14 days and had good validity in Chinese adolescents [25], with the same response options and scoring as the PHQ-9. The total score ranges from 0 to 21. Participants with higher scores in wave 2 were classified as having and the higher scores indicated more severe anxiety symptoms and anxiety symptoms were defined as GAD-7 scores ≥ 5 [26]. Cronbach’s alpha coefficients are 0.90 and 0.93 in waves 1 and 2, respectively, suggesting a high reliability.

#### Covariates

Covariates were selected as potential confounders based on previous literature [27, 28]. Covariates were obtained through questionnaires, including age, sex (1 = boy, 2 = girl), ethnicity (1 = Han 2 = others), academic pressure, smoking status, and drinking status. Except for age (continuous variables), all variables were included as categorical variables. Academic pressure was assessed by asking the participant’s self-rating of his or her academic pressure (1 = below average, 2 = average and 3 = above average). Current smoking status and drinking status were collected by asking the students “Have you used at least one cigarette during your lifetime?” and “Have you had at least one drink of alcohol during your lifetime?” Responses were coded as yes or no. We also created a directed acyclic graph (DAG) to visually represent the potential confounding relationships (eFigure 1) and determine the necessary adjustment sets of covariates for the multivariable analyses.

### Statistical analysis

Descriptive analyses were initially performed, and continuous and categorical variables were respectively expressed as mean (standard deviation, SD) and number (percentage, %). Participant characteristics were compared based on depression symptoms status (defined as a probable depression symptoms state with PHQ-9 scores ≥ 5) and anxiety symptoms status (defined as a probable anxiety symptoms state with GAD-7 scores ≥ 5) in wave 2 using two-sample t-tests for continuous variables or Chi-squared tests categorical variables. Additionally, we describe the distribution of participants’ age and sex (Table S3).

Generalized linear mixed-effects models were used to examine the associations of the changes in adherence to the 24-HMG with depression and anxiety symptoms. Considering the multi-stage sampling design and the potential difference in school culture and environment, the school unit was incorporated into the models as a random-effects term. The dependent variable in these models was the PHQ-9 scores or the GAD-7 scores, with the *β* coefficients and corresponding 95% CIs calculated, where a *β* coefficient > 0 indicated a positive association [29]. In all cases never meeting the guideline was the reference group. The variables identified as meeting the covariate criteria outlined in the DAG (eFigure 1), were included as control variables in the subsequent generalized linear mixed-effects models. Model 1 is unadjusted, and model 2 was adjusted for age, sex, academic pressure, smoking, and drinking status, depression symptoms status (only for depression symptoms), and anxiety symptoms status (only for anxiety symptoms) at baseline. Moreover, when examining the associations between changes in adherence to the individual guidelines and outcomes, we additionally adjusted the baseline levels of the other two movement behaviors in the models.

We investigated the associations of the changes in adherence to the 24-HMG with depression and anxiety symptoms in boys and girls, respectively, adjusting the above covariates. Besides, a sensitivity analysis was performed to verify the robustness of the results. In this sensitivity analysis, we further adjusted both baseline depression and anxiety symptoms scores in models to assess their effects on the results. The overall fits of all models were evaluated with the determinate coefficient (R^2^) to indicate the fraction of the variance that is explained by the model (Table S4) [30].

All analyses were performed using R 4.2.2 (The R Foundation for Statistical Computing, Vienna, Austria), and a two-sided *P*-value < 0.05 was considered statistically significant.

## Results

### Sample characteristics

We included 12,570 participants with a mean (SD) age of 13.0 (1.9) years among whom 6560 (52.2%) were boys and 6010 (47.8%) were girls (Table 1). The prevalence of depression symptoms and anxiety symptoms at follow-up were 39.9% and 37.2%, respectively. Adolescents with depression and anxiety symptoms were more likely to be older, girls, smoking and drinking, and having higher academic pressure than those without depression and anxiety symptoms.

**Table 1:** Characteristics of adolescents included in analyses

Variable^a^	All (n = 12,570)	Depressive symptoms			Anxiety symptoms		
		Yes (n = 5017)	No (n = 7553)	*P-*value ^b^	Yes (n = 4674)	No (n = 7904)	*P-*value^b^
Age, mean (SD), years	13.0 (1.9)	13.4 (1.8)	12.7 (1.9)	< 0.001	13.4 (1.8)	12.8 (1.9)	< 0.001
Sex							
Boys	6560 (52.2)	2200 (43.9)	4360 (57.7)	< 0.001	2027 (43.4)	4533 (57.4)	< 0.001
Girls	6010 (47.8)	2817 (56.1)	3193 (42.3)		2646 (56.6)	3364 (42.6)	
Ethnicity							
Han	11,888 (94.6)	4760 (94.9)	7128 (94.4)	0.460	4425 (94.7)	7463 (94.5)	0.899
Others	455 (3.6)	170 (3.4)	285 (3.8)		166 (3.6)	289 (3.7)	
Missing data	227 (1.8)	87 (1.7)	140 (1.9)		82 (1.8)	145 (1.8)	
Academic pressure							
Below average	3129 (24.9)	745 (14.8)	2384 (31.6)	< 0.001	645 (13.8)	2484 (31.5)	< 0.001
Average	7371 (58.6)	2976 (59.3)	4395 (58.2)		2785 (59.6)	4586 (58.1)	
Above average	1985 (15.7)	1267 (25.2)	718 (9.5)		1215 (26.0)	770 (9.8)	
Missing data	85 (0.7)	29 (0.6)	56 (0.7)		28 (0.6)	57 (0.7)	
Smoking status							
No	12,234 (97.3)	4846 (96.6)	7388 (97.8)	< 0.001	4519 (96.7)	7715 (97.7)	0.002
Yes	297 (2.4)	155 (3.1)	142 (1.9)		140 (3.0)	157 (2.0)	
Missing data	39 (0.3)	16 (0.3)	23 (0.3)		14 (0.3)	25 (0.3)	
Drinking status							
No	8381 (66.7)	2921 (58.2)	5460 (72.3)	< 0.001	2760 (59.1)	5621 (71.2)	< 0.001
Yes	4110 (32.7)	2067 (41.2)	2043 (27.0)		1884 (40.3)	2226 (28.2)	
Missing data	79 (0.6)	29 (0.6)	50 (0.7)		29 (0.6)	50 (0.6)	
Screen time guideline							
Persistent non-adherence	4907 (39.0)	2296 (45.8)	2611 (34.6)	< 0.001	2880 (61.6)	4315 (54.6)	< 0.001
Adherence to non-adherence	403 (3.2)	159 (3.2)	244 (3.2)		666 (14.3)	1272 (16.1)	
Non-adherence to adherence	6005 (47.8)	2190 (43.7)	3815 (50.5)		574 (12.3)	1111 (14.1)	
Persistent adherence	1255 (10.0)	372 (7.4)	883 (11.7)		553 (11.8)	1199 (15.2)	
Sleep duration guideline							
Persistent non-adherence	9357 (74.4)	4083 (81.4)	5274 (69.8)	< 0.001	3790 (81.1)	5567 (70.5)	< 0.001
Adherence to non-adherence	1279 (10.2)	431 (8.6)	848 (11.2)		409 (8.8)	870 (11.0)	
Non-adherence to adherence	1002 (8.0)	307 (6.1)	695 (9.2)		285 (6.1)	717 (9.1)	
Persistent adherence	932 (7.4)	196 (3.9)	736 (9.7)		189 (4.0)	743 (9.4)	
Physical activity guideline							
Persistent non-adherence	7195 (57.2)	3140 (62.6)	4055 (53.7)	< 0.001	2880 (61.6)	4315 (54.6)	< 0.001
Adherence to non-adherence	1938 (15.4)	715 (14.3)	1223 (16.2)		666 (14.3)	1272 (16.1)	
Non-adherence to adherence	1685 (13.4)	586 (11.7)	1099 (14.6)		574 (12.3)	1111 (14.1)	
Persistent adherence	1752 (13.9)	576 (11.5)	1176 (15.6)		553 (11.8)	1199 (15.2)	
Changes in the numbers of adherence to the 24-HMG (continuous), mean (SD)^c^	0.40 (0.88)	0.35 (0.84)	0.44 (0.91)	< 0.001	0.36 (0.85)	0.43 (0.90)	< 0.001
Changes in the numbers of adherence to the 24-HMG (categorical)							
Unchanged (scores = 0)	4972 (39.6)	2140 (42.7)	2832 (37.5)	< 0.001	1943 (41.6)	3029 (38.4)	< 0.001
Increasing (scores > 0)	5881 (46.8)	2199 (43.8)	3682 (48.7)		2082 (44.6)	3799 (48.1)	
Decreasing (scores < 0)	1717 (13.7)	678 (13.5)	1039 (13.8)		648 (13.9)	1069 (13.5)	
PHQ-9 scores at follow-up, mean (SD)^d^	4.63 (5.13)	9.59 (4.67)	1.33 (1.47)	< 0.001	8.96 (5.41)	2.06 (2.63)	< 0.001
GAD-7 scores at follow-up, mean (SD)^e^	4.08 (4.69)	7.81 (4.94)	1.61 (2.27)	< 0.001	8.98 (4.20)	1.18 (1.42)	< 0.001

*SD* standard deviation, *PHQ-9* the nine-item Patient Health Questionnaire, *GAD-7* the seven-item Generalized Anxiety Disorder Scale

^a^Unless otherwise indicated, data are expressed as No. (%) of participants

^b^Two-sample *t*-tests were used to compare the means of continuous variables. Pearson Chi-squared tests were performed to compare the distribution of categorical variables

^c^Scores range from − 3 to 3, with a higher score indicating that students met more movement guidelines in the follow-up compared to the baseline

^d^Scores range from 0 to 27, with higher scores indicating higher depression symptoms

^e^Scores range from 0 to 21, with higher scores indicating higher anxiety symptoms

### Changes in adherence to the 24-h movement guidelines and depression and anxiety symptoms

For changes in adherence to the individual guideline (Table 2), compared with persistent non-adherence to ST guideline, non-adherence to adherence (*β*, − 0.78; 95% CI − 1.08, − 0.48) and persistent adherence (*β*, − 0.85; 95% CI − 1.03, − 0.68) were associated with lower PHQ-9 scores even after adjusting for covariates in model 2, but adherence to non-adherence was not (*β*, 0.38; 95% CI − 0.09, 0.84). Compared with persistent non-adherence to the sleep duration guideline, non-adherence to adherence (*β*, − 0.80; 95% CI − 1.11, − 0.49) and persistent adherence (*β*, − 1.04; 95% CI − 1.37, − 0.70) were associated with lower PHQ-9 scores in model 2, but adherence to non-adherence was not (*β*, − 0.22, 95% CI − 0.50, 0.06). In contrast, changes in adherence to the physical activity guideline were not related to depression symptoms. The association of changes in adherence to the individual guidelines with anxiety symptoms was similar to the association of changes in adherence to the individual guidelines with depression symptoms.

**Table 2:** Association between changes in adherence to the 24-HMG with depression and anxiety symptoms

	N (%)	*β* (95% CI)^a^			
		Depressive symptoms		Anxiety symptoms	
		Model 1	Model 2	Model 1	Model 2
Screen time guideline					
Persistent non-adherence	4907 (39.0)	0 (Reference)	0 (Reference)	0 (Reference)	0 (Reference)
Adherence to non-adherence	403 (3.2)	− 0.04 (− 0.55, 0.48)	0.38 (− 0.09, 0.84)	0.10 (− 0.37, 0.57)	0.40 (− 0.03, 0.83)
Non-adherence to adherence	6005 (47.8)	**− 1.13 (− 1.33, − 0.94)**	**− 0.78 (− 1.08, − 0.48)**	**− 0.78 (− 0.95, − 0.60)**	**− 0.59 (− 0.76, − 0.43)**
Persistent adherence	1255 (10.0)	**− 1.62 (− 1.96, − 1.29)**	**− 0.85 (− 1.03, − 0.68)**	**− 1.18 (− 1.49, − 0.88)**	**− 0.59 (− 0.87, − 0.31)**
Sleep duration guideline					
Persistent non-adherence	9357 (74.4)	0 (Reference)	0 (Reference)	0 (Reference)	0 (Reference)
Adherence to non-adherence	1279 (10.2)	**− 0.76 (− 1.07, − 0.45)**	− 0.22 (− 0.50, 0.06)	**− 0.66 (− 0.94, − 0.37)**	− 0.24 (− 0.50, 0.02)
Non-adherence to adherence	1002 (8.0)	**− 1.22 (− 1.57, − 0.87)**	**− 0.80 (− 1.11, − 0.49)**	**− 1.09 (− 1.40, − 0.77)**	**− 0.69 (− 0.98, − 0.40)**
Persistent adherence	932 (7.4)	**− 1.88 (− 2.26, − 1.50)**	**− 1.04 (− 1.37, − 0.70)**	**− 1.61 (− 1.96, − 1.27)**	**− 0.82 (− 1.13, − 0.50)**
Physical activity guideline					
Persistent non-adherence	7195 (57.2)	0 (Reference)	0 (Reference)	0 (Reference)	0 (Reference)
Adherence to non-adherence	1938 (15.4)	**− 0.44 (− 0.69, − 0.18)**	− 0.03 (− 0.26, 0.20)	**− 0.31 (− 0.54, − 0.08)**	0.09 (− 0.13, 0.30)
Non-adherence to adherence	1685 (13.4)	**− 0.67 (− 0.94, − 0.40)**	− 0.16 (− 0.40, 0.09)	**− 0.54 (− 0.79, − 0.30)**	− 0.07 (− 0.30, 0.16)
Persistent adherence	1752 (13.9)	**− 0.82 (− 1.09, − 0.56)**	− 0.08 (− 0.33, 0.17)	**− 0.71 (− 0.96, − 0.47)**	− 0.09 (− 0.32, 0.14)
Changes in the numbers of adherence to 24-HMG (continuous)	–	**− 0.95 (− 1.07, − 0.83)**	**− 0.58 (− 0.69, − 0.47)**	**− 0.74 (− 0.85, − 0.63)**	**− 0.43 (− 0.53, − 0.33)**
Changes in the numbers of adherence to 24-HMG (categorical)					
Unchanged	4972 (39.6)	0 (Reference)	0 (Reference)	0 (Reference)	0 (Reference)
Increasing	5881 (46.8)	**− 1.16 (− 1.36, − 0.96)**	**− 0.70 (− 0.88, − 0.52)**	**− 0.87 (− 1.05, − 0.69)**	**− 0.50 (− 0.66, − 0.33)**
Decreasing	1717 (13.7)	**0.89 (0.59, 1.19)**	**0.55 (0.28, 0.82)**	**0.72 (0.44, 0.98)**	**0.44 (0.19, 0.69)**

Model 1 was the unadjusted model

Model 2 was adjusted for age, sex, academic performance, smoking, and drinking status, PHQ-9 scores (only for depression symptoms), and GAD-7 scores (only for anxiety symptoms) at baseline

*CI* confidence interval

^a^*β* coefficients and their 95% CIs were reported as unstandardized coefficients

For changes in the numbers of adherence to guidelines (Table 2), compared with the unchanged group, the increasing group showed lower levels of depression symptoms (*β*, − 0.70; 95% CI − 0.88, − 0.52) and anxiety symptoms (*β*, − 0.50; 95% CI − 0.66, − 0.33), but the decreasing group showed higher levels of depression symptoms (*β*, 0.55; 95% CI 0.28, 0.82) and anxiety symptoms (*β*, 0.44; 95% CI 0.19, 0.69). Moreover, each point increase in changes in the numbers of adherence to guidelines was found to be negatively associated with depression symptoms (*β*, − 0.58; 95% CI − 0.69, − 0.47) and anxiety symptoms (*β*, − 0.43; 95% CI − 0.53, − 0.33).

### Sex-stratified analyses

Similar patterns were observed in both boys and girls regarding the association between changes in guideline adherence and outcomes (Table 3). For anxiety symptoms, girls persistently adhering to sleep guidelines had a lower *β* coefficient (− 1.25, 95% CI − 1.79, − 0.71) than boys (− 0.59, 95% CI − 0.96, − 0.21) (*P* = 0.014). Furthermore, total adherence scores revealed significant sex differences in both depression and anxiety symptoms (depression symptoms: *P* = 0.004, anxiety symptoms: *P* = 0.014). In both boys and girls, meeting the physical activity guideline was still not associated with outcomes.

**Table 3:** Association between changes in adherence to the 24-HMG with depression and anxiety symptoms in different gender

	*β* (95% CI)^a^					
	Depression symptoms			Anxiety symptoms		
	Boys	Girls	*P* value^#^	Boys	Girls	*P* value^#^
Screen time guideline						
Persistent non-adherence	0 (Reference)	0 (Reference)	–	0 (Reference)	0 (Reference)	–
Adherence to non-adherence	0.50 (− 0.07, 1.07)	0.16 (− 0.60, 0.92)	0.238	0.37 (− 0.16, 0.90)	0.44 (− 0.27, 1.14)	0.440
Non-adherence to adherence	**− 0.64 (− 0.87, − 0.42)**	**− 0.86 (− 1.31, − 0.41)**	0.204	**− 0.46 (− 0.67, − 0.25)**	**− 0.60 (− 1.02, − 0.18)**	0.272
Persistent adherence	**− 0.71 (− 1.11, − 0.32)**	**− 1.07 (− 1.34, − 0.80)**	0.073	**− 0.56 (− 0.92, − 0.19)**	**− 0.71 (− 0.96, − 0.46)**	0.253
Sleep duration guideline						
Persistent non-adherence	0 (Reference)	0 (Reference)	–	0 (Reference)	0 (Reference)	–
Adherence to non-adherence	− 0.11 (− 0.46, 0.24)	− 0.34 (− 0.79, 0.10)	0.207	− 0.21 (− 0.54, 0.11)	− 0.25 (− 0.67, 0.16)	0.442
Non-adherence to adherence	**− 0.57 (− 0.95, − 0.19)**	**− 1.12 (− 1.66, − 0.56)**	0.055	**− 0.55 (− 0.90, − 0.19)**	**− 0.96 (− 1.43, − 0.49)**	0.086
Persistent adherence	**− 0.85 (− 1.25, − 0.45)**	**− 1.36 (− 1.94, − 0.78)**	0.070	**− 0.59 (− 0.96, − 0.21)**	**− 1.25 (− 1.79, − 0.71)**	**0.024**
Physical activity guideline						
Persistent non-adherence	0 (Reference)	0 (Reference)	–	0 (Reference)	0 (Reference)	–
Adherence to non-adherence	− 0.07 (− 0.36, 0.22)	0.06 (− 0.30, 0.43)	0.288	0.08 (− 0.20, 0.35)	0.13 (− 0.21, 0.47)	0.411
Non-adherence to adherence	− 0.21 (− 0.51, 0.09)	− 0.10 (− 0.51, 0.31)	0.331	− 0.08 (− 0.36, 0.20)	− 0.06 (− 0.44, 0.31)	0.468
Persistent adherence	0.02 (− 0.26, 0.30)	− 0.32 (− 0.83, 0.19)	0.123	− 0.04 (− 0.30, 0.22)	− 0.16 (− 0.63, 0.31)	0.336
Changes in the numbers of adherence to 24-HMG (continuous)	**− 0.45 (− 0.59, − 0.31)**	**− 0.76 (− 0.93, − 0.58)**	**0.004**	**− 0.33 (− 0.46, − 0.21)**	**− 0.57 (− 0.73, − 0.40)**	**0.014**
Changes in the numbers of adherence to 24-HMG (categorical)						
Unchanged	0 (Reference)	0 (Reference)	–	0 (Reference)	0 (Reference)	–
Increasing	**− 0.53 (− 0.77, − 0.30)**	**− 0.89 (− 1.16, − 0.62)**	0.339	**− 0.39 (− 0.61, − 0.17)**	**− 0.61 (− 0.86, − 0.36)**	0.471
Decreasing	**0.54 (0.21, 0.87)**	**0.60 (0.15, 1.05)**	0.311	**0.37 (0.07, 0.68)**	**0.55 (0.14, 0.97)**	0.442

All models were adjusted for age, sex, academic performance, smoking, and drinking status, PHQ-9 scores (only for depression symptoms), and GAD-7 scores (only for anxiety symptoms) at baseline

*CI* confidence interval

^#^The statistical significance of the differences between the strata was tested by using the 95% CI, one-side *P* value

^a^*β* coefficients and their 95% CIs were reported as unstandardized coefficients

### Sensitivity analysis

Similar results were obtained in the sensitivity analysis (Tables S1, S2).

## Discussion

Few studies have explored the association of changes in adherence to the 24-HMG with depression and anxiety symptoms among adolescents. In this longitudinal study, we found that changes in adherence to the 24-HMG were associated with depression and anxiety symptoms among Chinese adolescents. Specifically, transitioning from non-adherence to adherence and persistent adherence to ST and sleep duration guidelines were associated with lower levels of depression and anxiety symptoms, but changes in adherence to the physical activity guideline were not. Adolescents with increased adherence to the guidelines have lower levels of depression and anxiety symptoms. Sex differences were found in the association between persistently adhering to sleep guideline and anxiety symptoms and the associations between changes in the numbers of adherence to the 24-HMG adherence and depression and anxiety symptoms. Our findings provide a scientific basis for preventing depression and anxiety symptoms in adolescents.

We found that the prevalences of depression and anxiety symptoms were 39.9% and 37.2% at follow-up. These prevalences are higher compared to the previous review, which found a pooled prevalence of depression and anxiety symptoms of 25.2% and 20.5%, respectively, based on early pandemic data from 80,879 adolescents [2]. Another recent meta-analysis reported that the pooled prevalence of depressive and anxiety symptoms was both 31%, which is similar to our study [31]. A possible explanation could be that the prevalence of mental disorders also increased as the pandemic progressed. It is likely that prolonged disruptions to youths’ daily routines, academic pressure, and social interactions resulted in a compounding of mental health issues as the pandemic progressed [31]. Specifically, lockdown restrictions during the pandemic may have affected movement behaviors of adolescents, with adolescents being less active, spending more time on screens, and less time on sleep during confinement than before the pandemic [32, 33], which resulted in mental disorders.

Our results support the importance of meeting sleep and ST guidelines. Only a few studies have investigated the longitudinal association between changes in movement behaviors and depression and anxiety symptoms among adolescents. For instance, a longitudinal study conducted during the COVID-19 pandemic showed that increased ST was associated with more severe depression and anxiety symptoms [34]. However, this study only looked at changes in ST and physical activity and did not look at the whole 24-HMG. Another cohort study measured adherence to the 24-HMG over 1 year and depression symptoms in adolescents, finding that changes in adherence to sleep and ST guidelines were associated with lower depression symptoms [11]. Consistently, our study found that transitioning from non-adherence to adherence and persistent adherence to ST and sleep duration guidelines were associated with lower levels of depression and anxiety symptoms. As others have reported, sleep deprivation can cause daytime fatigue and daytime functioning, which affects mental health [35]. Similarly, prolonged ST is also associated with depression and anxiety symptoms among adolescents [36]. Higher ST could not only lead to attention difficulties and addictive ST behavior but may also trigger violent behaviors [37], all of which are recognized as significant risk factors for depression and anxiety symptoms.

We found no obvious association between changes in meeting physical activity guideline with depression and anxiety symptoms in the general population and both sexes. This finding seems to conflict with previous evidence, which suggests that physical activity interventions may help reduce depression symptoms in children and adolescents [38]. Nevertheless, the mechanism underlying the association between physical activity and mental health is likely complex. Mental health benefits may be more related to the context of physical activity. For example, participating in team sports and informal group activities was found to be associated with lower risks of mental health issues [39], indicating that it is the context of team sports rather than the volume of physical activity that provides mental health benefits. Indeed, our findings are consistent with previous studies suggesting that meeting the recommendations for ST and sleep duration, rather than physical activity, were associated with greater mental health benefits [3]. Nonetheless, meeting physical activity recommendation should not be neglected, as adherence to meeting more recommendations was associated with lower risks of depression and anxiety symptoms, as mentioned above. Future research should consider the context and types of physical activities to gain a deeper understanding of how physical activities influence depression and anxiety symptoms in adolescents.

For the transition in total guideline adherence, meeting more guidelines predicted lower depression and anxiety symptoms, indicating a dose–response relationship between the number of meeting recommendations and psychological health outcomes, consistent with previous research [40]. A cohort study conducted in Canada with 2292 adolescents in grades 9–12 reported that meeting additional guidelines was associated with lower depressive symptoms among girls only [11]. Another cohort study reported that meeting more guidelines was not associated with higher flourishing [22]. The relatively small sample size (< 3000), lack of adjustment for potential confounding factors (i.e., smoking and drinking status), and differences in the outcome may lead to inconsistent findings in previous studies. Our study confirmed previous findings on the dynamic nature of adherence to the 24-HMG among adolescents. In addition, our study extends previous findings by revealing the association between changes in adherence to the 24-HMG with anxiety symptoms. From the results, it is crucial to adhere to guidelines consistently to prevent depression and anxiety symptoms. Making timely adjustments to adolescents’ lifestyles can help reduce the risks of experiencing depression and anxiety symptoms.

In this study, we found girls with persistently adhering to sleep guidelines had lower anxiety symptoms than boys. Changes in the number of adherence to overall 24-HMG also had significant sex differences in both depression and anxiety symptoms. These findings are consistent with previous studies [11, 41]. These differences may be attributed to the higher levels of depression and anxiety symptoms typically observed in girls [17]. Evidence has shown that girls’ emotional recognition and the biological stress response are more negatively affected by sleep restriction [42, 43]. Persistently adhering to sleep guideline may help girls reduce stress more effectively, reducing anxiety symptoms. However, in this study, no significant sex differences were found in changes in adherence to ST guidelines, which was consistent with a previous study [44]. While some previous studies suggested that boys may be more likely to play video games [45] and girls are more likely to be involved in more social media use [46], these differences may be diminishing in the same school environment, leading to similar screen time behaviors. Therefore, we did not observe sex differences in associations between changes in the adherence to ST guidelines and depression and anxiety symptoms. These findings indicated that both boys and girls benefit equally from meeting this guideline. This suggests that while there may be some sex differences in some areas, overall adherence to the 24-HMG is important for preventing depression and anxiety symptoms in all adolescents, regardless of sex.

These findings may raise some enlightenments for enhancing mental health among adolescents. Firstly, our findings provide essential information to better understand the associations between the 24-HMG and depression and anxiety symptoms. Future public health strategies that aim to promote adolescents’ mental health are highly recommended to target reducing ST and increasing sleep duration simultaneously both in school and community settings. Furthermore, movement behaviors are modifiable, and decreased depression and anxiety symptoms were observed in adolescents who transitioning from non-adherence to adherence to ST and sleep guidelines. Therefore, many interventions are needed to encourage adolescents to enhance healthy movement behaviors to meet more guidelines of the 24-HMG, especially ST and sleep. School health education on sufficient sleep and advisable ST should be provided to help adolescents realize healthy lifestyle behaviors. Finally, interventions should include early identification and intervention strategies for adolescent internet addiction to prevent adolescents’ excessive ST.

The strengths of our study include the longitudinal design, the large sample, and the consideration of changes in adherence to the 24-HMG. However, there are some limitations. First, we used questionnaires to collect data, which may introduce recall bias and reporting bias. It is necessary to use computer technology to objectively and accurately collect data. Second, we acknowledge that the determinate coefficients for some models are relatively low, indicating that a significant portion of the variance in the outcome remains unexplained by the included covariates. However, because of the nature of this observational study, the results may be influenced by unknown or unmeasured confounding factor(s). Additionally, the primary aim of our study was not to maximize predictive power but to explore associations between key variables. Finally, due to the short follow-up period in our study, a longer-term cohort study is necessary to confirm our findings. Moreover, only two waves of data were available at the time of this analysis. Future research should thus include more time points and longer intervals will help to improve measurement reliability and accuracy.

## Conclusions

In this longitudinal study, non-adherence to adherence and persistent adherence to ST and sleep duration guidelines were associated with lower levels of depression and anxiety symptoms, but changes in adherence to physical activity guideline were not related to outcomes. Adolescents with increased adherence to the guidelines had lower depression and anxiety symptoms. The association of persistently adhering to sleep guideline with anxiety symptoms and the associations of changes in the numbers of adherence to the 24-HMG had sex differences. Our findings suggest that adolescents should be encouraged to maintain and enhance healthy movement behaviors to meet more guidelines of the 24-HMG, especially ST and sleep. Parents and schools are highly recommended to address ST and sleep together as crucial components for depression and anxiety symptoms prevention. Specifically, health education on sleep and ST and early identification and intervention strategies for adolescent internet addiction ST should be provided. Moreover, it is essential to conduct longer-term cohort studies and randomized controlled trials to validate our findings.

## Supplementary Information

Supplementary Material 1.

## Data Availability

No datasets were generated or analysed during the current study.

## Abbreviations

SD: Standard deviation
CI: Confidence interval
PHQ-9: 9-Item version of Patient Health Questionnaire
GAD-7: 7-Item version of General Anxiety Disorder Scale
24-HMG: 24-Hour movement guidelines
ST: Screen time
MVPA: Moderate-to-vigorous physical activity
