# Supplementary material for: Association between changes in adherence to the 24-hour movement guidelines with depression and anxiety symptoms among Chinese adolescents: a prospective population-based study
Source: Child Adolesc Psychiatry Ment Health. 2024 Nov 10;18:143. doi: 10.1186/s13034-024-00836-7 (PMC11550551; doi:10.1186/s13034-024-00836-7)
Supplement: Supplementary file 1 — Supplementary Material 1. [file 13034_2024_836_MOESM1_ESM.docx]

**Association between changes in adherence to the 24-hour movement guidelines with depressive and anxiety symptoms among Chinese adolescents: a prospective population-based study**

Herui Wu, Master ^a, #^, Yi-fan Lin, PhD ^b, c, #^, Liwen Yang, Master ^a^, Wenjian Lai, PhD ^a^, Yanzhi Li, PhD ^a^, Ye Xu, Master ^a^, Wanxin Wang, PhD ^a^, Lei Yang ^b^, PhD, Ciyong Lu, PhD ^a, ‡, *^, Bin Yan, Master ^b, c, ‡, *^

**Author Affiliations:**

^a^ Department of Medical Statistics and Epidemiology, School of Public Health, Sun Yat-sen University, Guangzhou, China

^b^ The First Affiliated Hospital, Shenzhen University, Shenzhen Second People’s Hospital, Shenzhen, Guangdong, China

^c^ Department of spine surgery, the First Affiliated Hospital, Shenzhen University, Shenzhen Second People’s Hospital, Shenzhen, Guangdong, China.

^#^ These authors contributed equally to this work.

^‡^ These authors contributed equally to this work.

***Corresponding authors**

Ciyong Lu

Department of Medical Statistics and Epidemiology, School of Public Health, Sun Yat-sen University, 74 Zhongshan Rd 2, Guangzhou, Guangdong 510080, China

Tel: (8620) 87332477, Email: luciyong@mail.sysu.edu.cn

Bin Yan

Department of spine surgery

the Fist Affiliated Hospital of Shenzhen University

Number 3002, Sungang west road, Futian district, Shenzhen 518035, China

Tel: (0755) 82557925, E-mail: [yanbinzhiyou@163.com](mailto:yanbinzhiyou@163.com)

**Supplementary Material**

**eFigure 1.** Directed acyclic graph of the potential confounding relationships between covariates.

**Table S1.** Association between changes in adherence to physical activity, screen time, and sleep duration guidelines and all three guidelines with depression symptoms and anxiety symptoms adjusting GAD-7 scores and PHQ-9 scores at baseline.

**Table S2.** Association between transitions of physical activity, screen time, and sleep duration guidelines and all three guidelines with depression symptoms and anxiety symptoms in different genders adjusting GAD-7 scores and PHQ-9 scores at baseline.

**Table S3.** Age Distribution of he analyzed study sample.

**Table S4.** The determinate coefficient (R^2^) of associations between changes in adherence to the 24-HMG with depression and anxiety symptoms.

**eFigure 1.** Directed acyclic graph of the potential confounding relationships between covariates.


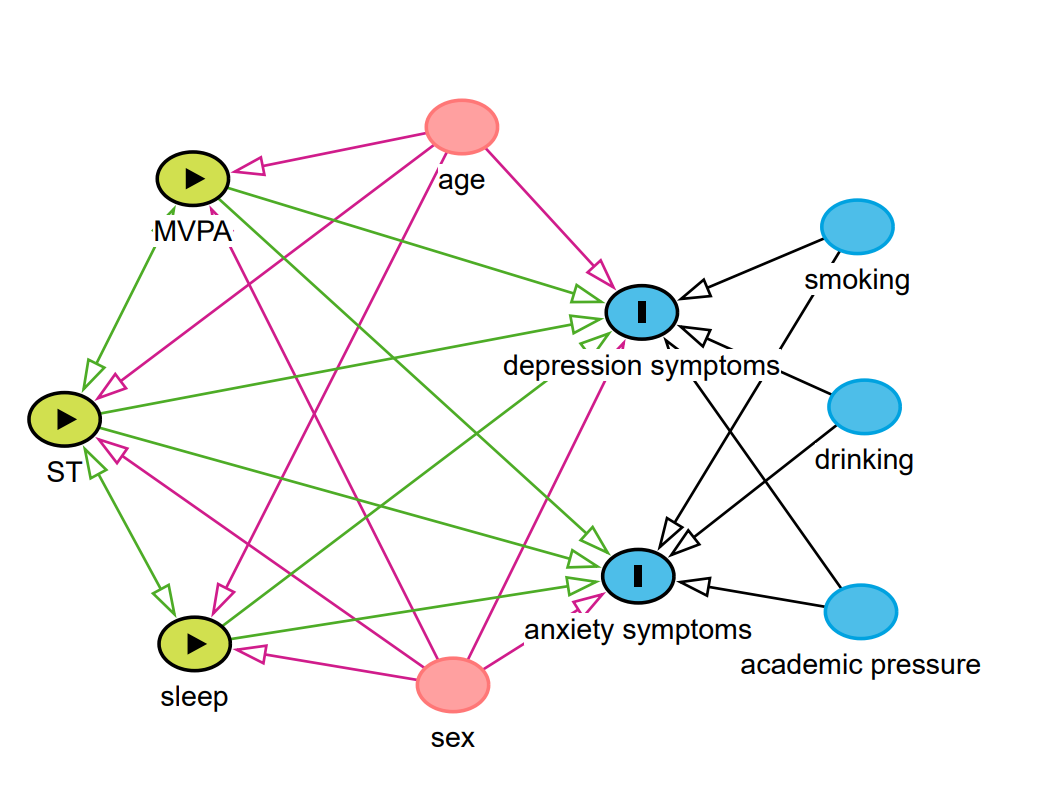


We generated this figure using DAGitty software, version 3.0[1-3].

ST; screen time

MVPA; moderate-to-vigorous physical activity

| **Table S1. Association between changes in adherence to physical activity, screen time, and sleep duration guidelines and all three guidelines with depression symptoms and anxiety symptoms adjusting GAD-7 scores and PHQ-9 scores at baseline.** | | |
| --- | --- | --- |
|  | ***β* (95%CI) ^*^** | |
|  | **Depressive Symptoms** | **Anxiety Symptoms** |
| **Screen time guideline** |  |  |
| Persistent non-adherence | 0 (Reference) | 0 (Reference) |
| Adherence to non-adherence | 0.39 (-0.06, 0.85) | 0.44 (0.02, 0.86) |
| Non-adherence to adherence | **-0.82 (-1.00, -0.65)** | **-0.54 (-0.70, -0.38)** |
| Persistent adherence | **-0.72 (-1.01, -0.42)** | **-0.45 (-0.73, -0.18)** |
| **Sleep duration guideline** |  |  |
| Persistent non-adherence | 0 (Reference) | 0 (Reference) |
| Adherence to non-adherence | -0.18 (-0.46, 0.09) | -0.17 (-0.42, 0.09) |
| Non-adherence to adherence | **-0.75 (-1.05, -0.44)** | **-0.67 (-0.95, -0.39)** |
| Persistent adherence | **-0.94 (-1.27, -0.61)** | **-0.76 (-1.07, -0.45)** |
| **Physical activity guideline** |  |  |
| Persistent non-adherence | 0 (Reference) | 0 (Reference) |
| Adherence to non-adherence | 0.00 (-0.23, 0.23) | 0.12 (-0.09, 0.33) |
| Non-adherence to adherence | -0.14 (-0.38, 0.10) | -0.03 (-0.26, 0.19) |
| Persistent adherence | -0.08 (-0.33, 0.16) | -0.01 (-0.24, 0.22) |
| **Changes in the numbers of adherence to 24-HMG (continuous)** | **-0.56 (-0.67, -0.45)** | **-0.40 (-0.50, -0.30)** |
| **Changes in the numbers of adherence to 24-HMG (categorical)** |  |  |
| Unchanged | 0 (Reference) | 0 (Reference) |
| Increasing | **-0.69 (-0.86, -0.51)** | **-0.45 (-0.62, -0.29)** |
| Decreasing | **0.53 (0.26, 0.79)** | **0.41 (0.17, 0.66)** |

Abbreviation: CI, confidence interval

The model was adjusted for age, ethnicity, academic pressure, smoking and drinking status, depression symptoms status and anxiety symptoms status at baseline.

* *β* coefficient and its 95% CI were reported as unstandardized coefficients.

| **Table S2. Association between transitions of physical activity, screen time, and sleep duration guidelines and all three guidelines with depression symptoms and anxiety symptoms in different genders adjusting GAD-7 scores and PHQ-9 scores at baseline.** | | | | | | |
| --- | --- | --- | --- | --- | --- | --- |
|  | ***β* (95%CI) ^*^** | | | | | |
|  | **Depression symptoms** | | | **Anxiety symptoms** | | |
|  | **Boys** | **Girls** | ***P* value^#^** | **Boys** | **Girls** | ***P* value^#^** |
| **Screen time guideline** |  |  |  |  |  |  |
| Persistent non-adherence | 0 (Reference) | 0 (Reference) | - | 0 (Reference) | 0 (Reference) | - |
| Adherence to non-adherence | 0.48 (-0.08, 1.03) | 0.21 (-0.53, 0.96) | 0.289 | 0.43 (-0.09, 0.95) | 0.48 (-0.21, 1.16) | 0.459 |
| Non-adherence to adherence | **-0.68 (-0.90, -0.46)** | **-0.75 (-1.19, -0.31)** | 0.384 | **-0.44 (-0.65, -0.23)** | **-0.41 (-0.82, -0.01)** | 0.438 |
| Persistent adherence | **-0.71 (-1.09, -0.32)** | **-0.95 (-1.21, -0.69)** | 0.153 | **-0.46 (-0.82, -0.10)** | **-0.59 (-0.83, -0.35)** | 0.173 |
| **Sleep duration guideline** |  |  |  |  |  |  |
| Persistent non-adherence | 0 (Reference) | 0 (Reference) | - | 0 (Reference) | 0 (Reference) | - |
| Adherence to non-adherence | -0.05 (-0.39, 0.29) | -0.20 (-0.63, 0.23) | 0.296 | -0.08 (-0.40, 0.23) | -0.08 (-0.48, 0.32) | 0.495 |
| Non-adherence to adherence | **-0.48 (-0.85, -0.11)** | **-1.02 (-1.58, -0.47)** | 0.054 | **-0.43 (-0.78, -0.09)** | **-0.90 (-1.36, -0.45)** | 0.055 |
| Persistent adherence | **-0.67 (-1.06, -0.28)** | **-1.16 (-1.73, -0.59)** | 0.081 | **-0.5 (-0.87, -0.14)** | **-1.00 (-1.52, -0.48)** | 0.063 |
| **Physical activity guideline** |  |  |  |  |  |  |
| Persistent non-adherence | 0 (Reference) | 0 (Reference) | - | 0 (Reference) | 0 (Reference) | - |
| Adherence to non-adherence | 0.00 (-0.29, 0.28) | 0.08 (-0.27, 0.44) | 0.356 | 0.14 (-0.13, 0.40) | 0.18 (-0.14, 0.51) | 0.418 |
| Non-adherence to adherence | -0.20 (-0.49, 0.10) | -0.07 (-0.47, 0.33) | 0.308 | -0.03 (-0.31, 0.24) | -0.06 (-0.42, 0.31) | 0.462 |
| Persistent adherence | 0.04 (-0.23, 0.31) | -0.24 (-0.73, 0.25) | 0.161 | 0.09 (-0.17, 0.34) | -0.04 (-0.49, 0.41) | 0.313 |
| **Changes in the numbers of adherence to 24-HMG (continuous)** | **-0.44 (-0.58, -0.31)** | **-0.77 (-0.95, -0.60)** | **0.002** | **-0.30 (-0.43, -0.18)** | **-0.56 (-0.72, -0.39)** | **0.008** |
| **Changes in the numbers of adherence to 24-HMG (categorical)** |  |  |  |  |  |  |
| Unchanged | 0 (Reference) | 0 (Reference) | - | 0 (Reference) | 0 (Reference) | - |
| Increasing | **-0.53 (-0.77, -0.30)** | **-0.86 (-1.13, -0.59)** | **0.034** | **-0.36 (-0.58, -0.14)** | **0.53 (0.12, 0.94)** | 0.127 |
| **Table S2. Association between transitions of physical activity, screen time, and sleep duration guidelines and all three guidelines with depression symptoms and anxiety symptoms in different genders adjusting GAD-7 scores and PHQ-9 scores at baseline.** | | | | | | |
|  | ***β* (95%CI) ^*^** | | | | | |
|  | **Depression symptoms** | | | **Depression symptoms** | | |
|  | **Boys** | **Girls** | ***P* value^#^** | **Boys** | **Girls** | ***P* value^#^** |
| Decreasing | **0.53 (0.20, 0.85)** | **0.56 (0.11, 1.00)** | 0.456 | **0.34 (0.04, 0.65)** | **-0.33 (-0.56, -0.11)** | 0.240 |

Abbreviation: CI, confidence interval

All models were adjusted for age, sex, academic performance, smoking and drinking status, PHQ-9 scores and GAD-7 scores at baseline.

^#^ The statistical significance of the differences between the strata was tested by using the 95 % CI, one-side *P* value.

^*^ *β* coefficient and its 95% CI were reported as unstandardized coefficients.

| **Table S3. Age and sex distribution of he analyzed study sample** | |
| --- | --- |
| **Variable** | **All (n=12570)** |
| **Sex** |  |
| Boys | 6560 (52.2) |
| Girls | 6010 (47.8) |
| **Age** |  |
| <10 | 1821 (14.5) |
| 10-12 | 2722 (21.7) |
| 12-14 | 3708 (29.5) |
| 14-16 | 3203 (25.5) |
| >16 | 1116 (8.9) |

| **Table S4. The determinate coefficients (R^2^) of associations between changes in adherence to the 24-HMG with depression and anxiety symptoms.** | | |
| --- | --- | --- |
|  | **Depressive Symptoms** | **Anxiety Symptoms** |
| Screen time guideline | 0.356 | 0.334 |
| Sleep duration guideline | 0.349 | 0.339 |
| Physical activity guideline | 0.347 | 0.338 |
| Changes in the numbers of adherence to the 24-HMG (continuous) | 0.373 | 0.354 |
| Changes in the numbers of adherence to the 24-HMG (categorical) | 0.357 | 0.328 |

**References:**

1. Shrier I, Platt RW: Reducing bias through directed acyclic graphs. *BMC MED RES METHODOL* 2008, 8:70.

2. Steare T, Gutiérrez Muñoz C, Sullivan A, Lewis G: The association between academic pressure and adolescent mental health problems: A systematic review. *J AFFECT DISORDERS* 2023, 339:302-317.

3. Cao R, Gao T, Ren H, Hu Y, Qin Z, Liang L, Li C, Mei S: Unique and cumulative effects of lifestyle-related behaviors on depressive symptoms among Chinese adolescents. *The International journal of social psychiatry* 2022, 68(2):354-364.
